# Screening for nonalcoholic steatohepatitis by using cytokeratin 18 and transient elastography in HIV mono-infection

**DOI:** 10.1371/journal.pone.0191985

**Published:** 2018-01-30

**Authors:** Amine Benmassaoud, Peter Ghali, Joseph Cox, Philip Wong, Jason Szabo, Marc Deschenes, Maria Osikowicz, Bertrand Lebouche, Marina B. Klein, Giada Sebastiani

**Affiliations:** 1 Division of Gastroenterology and Hepatology, Royal Victoria Hospital, McGill University Health Centre, Montreal, Canada; 2 Chronic Viral Illness Service, McGill University Health Centre, Montreal, Canada; Kaohsiung Medical University, TAIWAN

## Abstract

**Background and aim:**

HIV-infected individuals are at high risk of developing nonalcoholic steatohepatitis (NASH), a leading cause of end-stage liver disease in Western countries. Nonetheless, due to the invasiveness of liver biopsy, NASH remains poorly understood in HIV mono-infection. We aimed to characterize the prevalence and predictors of NASH in unselected HIV mono-infected patients by means of non-invasive diagnostic tools.

**Methods:**

HIV-infected adults without significant alcohol intake or co-infection with hepatitis B or C underwent a routine screening program employing transient elastography (TE) with controlled attenuation parameter (CAP) and the serum biomarker cytokeratin-18 (CK-18). NASH was diagnosed non-invasively as the coexistence of fatty liver (CAP ≥248 dB/m) and CK-18 >246 U/L. Identified cases of NASH were offered a diagnostic liver biopsy. Predictors of NASH were determined by multivariate logistic regression analysis.

**Results:**

202 consecutive HIV mono-infected patients were included. NASH was non-invasively diagnosed in 23 cases (11.4%). Among them, 17 underwent a liver biopsy, and histology confirmed NASH in all cases. The prevalence of NASH was higher in patients with hypertriglyceridemia (17.1%), insulin resistance defined by homeostasis model for assessment of insulin resistance (HOMA-IR) (25%), those with detectable HIV viral load (42.9%) and those with elevated ALT (53.6%). After adjustment, higher HOMA-IR (adjusted odds ratio [aOR] = 1.20, 95% CI 1.01–1.43; p = 0.03) and ALT (aOR = 2.39, 95% CI 1.50–3.79; p<0.001) were independent predictors of NASH.

**Conclusions:**

NASH, diagnosed by a non-invasive diagnostic approach employing CK-18 and TE with CAP, is common in unselected HIV mono-infected individuals, particularly in the presence of insulin resistance and elevated ALT.

## Introduction

Liver disease is the leading cause of non-AIDS related deaths in people living with human immunodeficiency virus (HIV)[1]. Although this excess morbidity is mainly driven by co-infections with hepatitis B or C virus, nonalcoholic fatty liver disease (NAFLD) is increasingly being recognized as a common cause of liver fibrosis in HIV mono-infected patients receiving antiretroviral therapy (ART). In North America, the prevalence of NAFLD in the general population is estimated to be about 25%, compared to 50% in the HIV mono-infected population[2, 3]. HIV-infected individuals have unique risk factors for NAFLD, including long-term exposure to ART, dyslipidemia, and a high frequency of insulin resistance[4, 5]. Simple NAFLD is the first pathophysiological step leading to nonalcoholic steatohepatitis (NASH), a state of hepatocellular inflammation and damage in response to accumulated fat within the liver parenchyma. This process can then lead to cirrhosis, hepatocellular carcinoma (HCC) and liver failure[6]. NASH occurs in 3–5% of the general population, and it is a frequent indication for liver transplantation in Western countries[2, 7, 8]. NASH may affect up to 55% of HIV mono-infected patients on ART with chronic elevation of transaminases[9]. However, its true prevalence is unknown.

Liver biopsy is the gold standard to distinguish NASH from simple NAFLD and to stage liver fibrosis. However, it is invasive and serious complications occur in 0.6–5% of patients[10]. Moreover, liver biopsy is costly and prone to sampling errors leading to the misdiagnosis of cirrhosis[8, 11]. As such, it is not practical to be used as a screening tool in a population like HIV-infected patients, where the prevalence of the disease is potentially very high[3, 9, 12]. The liver stiffness measurement (LSM) by transient elastography (TE) is a validated non-invasive method to diagnose liver fibrosis, with a reported area under the curve (AUC) of 0.93 in HIV mono-infected patients[13]. Controlled Attenuation Parameter (CAP) is a TE software able to quantify fat in the liver with high diagnostic accuracy. A recent meta-analysis showed that CAP had an AUC of 0.82 for the detection of hepatic steatosis involving >10% of hepatocytes[14]. However, TE with CAP cannot differentiate between simple NAFLD and NASH. Cytokeratin-18 (CK-18) is a marker of hepatocyte apoptosis, which occurs in NASH but not in NAFLD[15]. CK-18 is the most validated diagnostic biomarker for NASH. Only one study so far has employed CK-18 in HIV mono-infected patients. However, this study was conducted in Asian patients, where NASH has different clinical characteristics[16]. No data about CK-18 is available in HIV mono-infected patients from Western countries.

In a clinical cohort of consecutive and unselected HIV mono-infected patients without known liver disease, we ascertained the prevalence and predictors of NASH diagnosed by combining CK-18 and TE with CAP.

## Patients and methods

### Study design and population

This was a single centre cross-sectional analysis of a prospective cohort of HIV-infected patients followed at the Chronic Viral Illness Service of the McGill University Health Centre (MUHC), a university-based clinic serving over 2,000 active HIV-infected patients. We included 202 consecutive HIV mono-infected individuals (positive enzyme-linked immunosorbent assay [ELISA] with Western blot confirmation), who underwent TE examination and determination of CK-18 between January 2015 and January 2017, as part of a routine screening program. In order to be included, patients had to fulfill the following criteria: (a) age ≥18 years; (b) availability of relevant clinical and biochemical parameters. Exclusion criteria were: (a) positivity for hepatitis C virus antibody; (b) positivity for hepatitis B surface antigen; (c) evidence of other liver diseases (e.g., auto-immune hepatitis, hemochromatosis, Wilson’s disease); (d) significant alcohol intake as measured by the Alcohol Use Disorders Identification Test (AUDIT-C) questionnaire, with a score equal or over 7 being excluded[17]; (e) current or past history of HCC; (f) prior liver transplantation; (g) failure of TE examination or unreliable LSM. All patients provided written informed consent. The Research Ethics Board of the Research Institute of MUHC approved the study (study code 14-182-BMD), which was conducted according to the Declaration of Helsinki.

### Outcome measures

The primary outcome was to determine the prevalence and predictors of NASH. Secondary outcomes aimed at evaluating factors correlating with CK-18 levels and the prevalence of NAFLD, significant liver fibrosis, and cirrhosis. Based on two meta-analyses and a study which specifically validated TE in HIV mono-infection, NASH was defined by the contemporaneous presence of NAFLD (hepatic steatosis involving >10% of hepatocytes), diagnosed by CAP ≥248 decibel/meter (dB/m) and CK-18 >246 U/L; significant liver fibrosis and cirrhosis were defined as LSM ≥7.1 kiloPascals (kPa) and ≥13kPa, respectively[3, 12–14]. The threshold used to define significant liver fibrosis was histological stage ≥2 out of 4 by the Brunt staging system (F2-4), while the threshold for cirrhosis was stage 4 out of 4 (F4)[18].

### TE with CAP examination

The examination was performed after 4-hours fasting by patients. The same experienced operator (**>**500 examinations before the study) performed all TE examinations as per manufacturer’s specifications. The standard M probe was used in all patients. The XL probe was used in case of failure with M probe and if body mass index (BMI) >30 Kg/m^2^. Examinations with no successful measurements after at least 10 attempts were deemed failures. The following criteria were applied to define the result of the examination as reliable: at least 10 validated measures, an interquartile range (IQR) <30% of the median, and >60% success rate[19]. Patients with known risk factors for a false positive LSM were also excluded[19]. The thresholds for liver fibrosis were decreased by -1.5 kPa to interpret the result with the XL probe[20]. Given recent data on the effect of severe steatosis on LSM, we also analyzed the number of cases at risk for false positivity due to elevated CAP (>300 dB/m)[21].

### CK-18 determination and diagnosis of NASH

A blood sample was obtained in all patients consecutively enrolled. The plasma was stored at -80°C until used for quantitative measurement of CK-18 levels by the Human cytokeratin ELISA kit (MJS Biolynx inc, Brockville Ontario, Canada).

### Histological assessment

Patients with a non-invasive diagnosis of NASH were offered a diagnostic percutaneous liver biopsy, as per standard of care. All biopsies were obtained with 16G Tru-Cut type needle. Liver biopsies were fixed in formalin and embedded in paraffin. The slides were stained with hematoxylin–eosin, Van Gieson’s stain for collagen, PAS- after diastase digestion, and Perls’ Prussian blue. All liver biopsies were interpreted by two experienced pathologists. The stage of fibrosis and degree of steatosis were reported according to the Brunt classification[18]. A diagnosis of NASH was made by the presence of classic histological features including steatosis, lobular inflammation, and ballooning[18].

### Clinical and biological parameters

Clinical parameters included age, gender, ethnicity, body mass index (BMI), history of type 2 diabetes mellitus, risk factors for HIV infection, time since HIV diagnosis, detailed history of ART (grouped by class), and alcohol intake. The diagnosis of diabetes was based on the definition of the International Diabetes Federation or the use of antidiabetic drugs[22]. Biological parameters were collected within 6 months of LSM and included: CD4 count, HIV viral load (COBAS Amplicor with lower limit of detection of 40 copies/mL), aspartate (AST) and alanine aminotransferases (ALT), gamma-glutamyl transpeptidase (GGT), platelet count, total cholesterol, low-density lipoprotein cholesterol (LDL), high-density lipoprotein cholesterol (HDL), triglycerides, fasting plasma glucose and insulin. Insulin resistance was determined using the homeostasis model for assessment of insulin resistance (HOMA-IR) index [fasting insulin (mIU/l) X fasting glucose (mmol/l) / 22.5][23]. Insulin resistance was defined as HOMA-IR ≥ 2, a cut-off point indicative of insulin resistance in other analyses[24]. The simple fibrosis biomarker AST-to-Platelets Ratio Index (APRI) was also calculated as follows: [100 x (AST/upper limit of normality)/platelet count (10^9^/L)[25].

### Statistical analysis

Continuous variables were expressed as mean (standard deviation [SD]), and categorical variables were presented as numbers (proportions). We compared characteristics of participants by outcome status using Student’s T test for continuous variables and Pearson’s chi-squared or Fisher's exact test for categorical variables. Correlation coefficients (r) were calculated using the Spearman rank correlation analysis. Predictors of NASH were determined using logistic regression analysis, which included covariates that were determined *a priori* to be clinically important. Results were reported as adjusted odds ratio (OR) with 95% confidence interval (CI). A complete case analysis was used for the multivariate models and the percentage of missing data was less than 10%, unless otherwise specified. To establish which of the models had the best goodness-of-fit measure, the corrected Akaike information criteria (AIC) and the Bayesian information criteria (BIC) were calculated and compared among the models using the ‘*estat*’ command in STATA. A lower AIC and/or BIC indicated a better fit. All tests were two-tailed and with a significance level of α = 0.05. Statistical analyses were performed using STATA 13.1.

## Results

**Fig 1:**
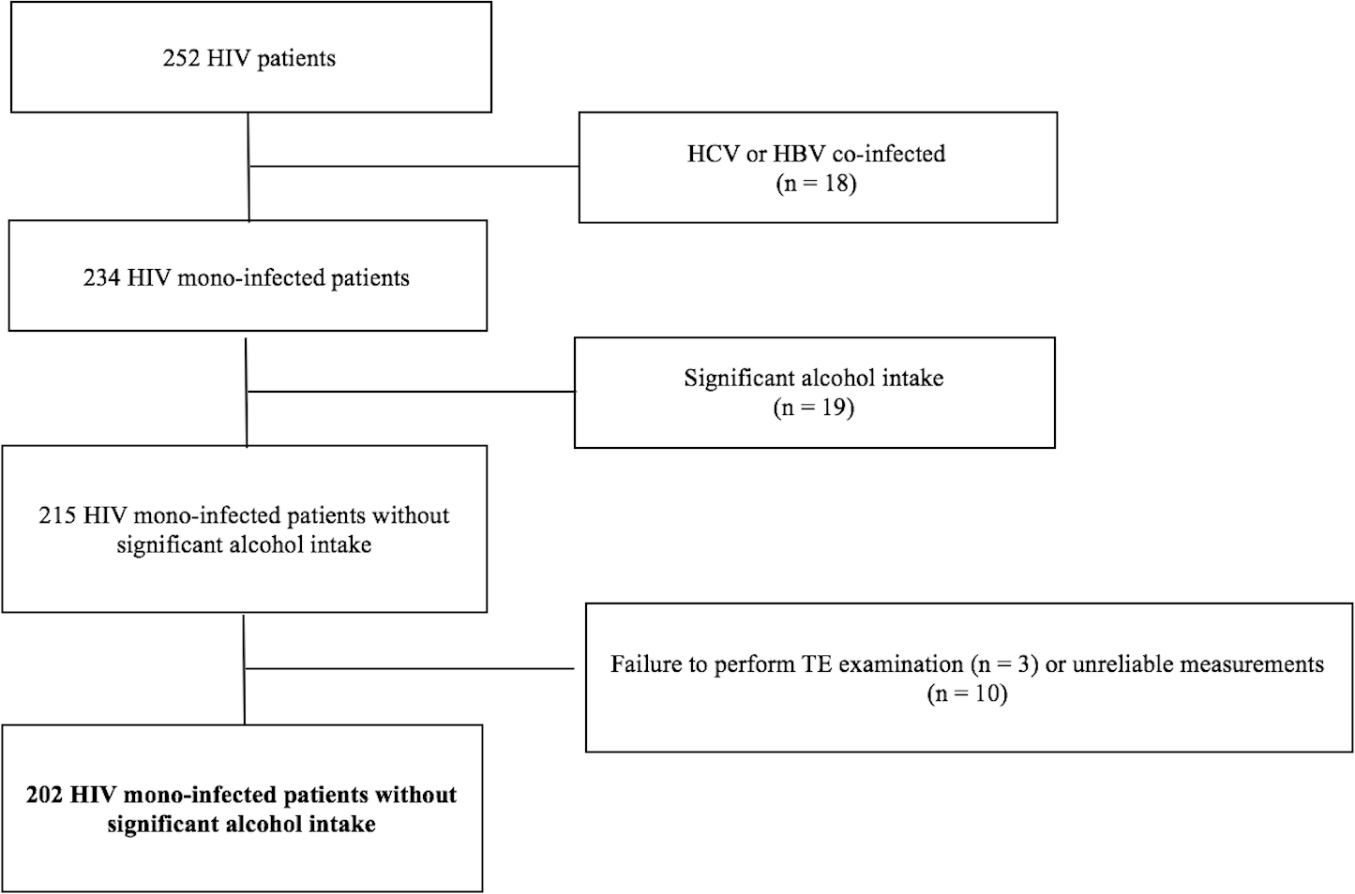
Flow chart displaying selection of study participants for analysis. Of 252 consecutive HIV patients who had a TE examination done at the Chronic Viral Illness Service and had available lab data, 18 were excluded because they were co-infected with HCV or HBV, 19 because of significant alcohol intake, 3 because of failure to perform TE examination and 10 because of unreliable measurements. Abbreviations: HIV, human immunodeficiency virus; HCV, hepatitis C virus; HBV, hepatitis B virus; TE, transient elastography.

After applying our inclusion and exclusion criteria (Fig 1), 202 patients with HIV mono-infection and without evidence of other liver diseases were included. The XL probe was used in 67 (33.2%) cases, while the standard M probe was applied in the remaining patients. Our TE failure rate (5.2%) was in line with previous studies[19]. Thirty-three cases (16.3%) had CAP >300 dB/m but only 6 (2.9%) had a LSM in the range of values that Petta *et al* reported to be at risk for false positivity[21]. The main demographic, clinical, biochemical and virological characteristics of the study population are summarized in Table 1. There were 157 (77.7%) males, and the mean age was 53.8 (SD 10.5) years. The most represented ethnicities were White/Caucasian and Black non-Hispanic. The main risk factor for HIV infection was men having sex with men. Obesity, defined as BMI ≥30kg/m^2^, was found in 57 (28.2%) cases. Insulin resistance, expressed by HOMA-IR ≥2, was found in 86 (61.4%) out of 140 cases where it was available. Overall, 109 (54.0%) patients had NAFLD, 22 (10.9%) patients had significant liver fibrosis, and 9 (4.5%) had cirrhosis.

**Table 1:** Demographic, clinical, biochemical, and virological characteristics of 202 patients with HIV mono-infection and univariate analysis by presence of NAFLD and NASH.

Variable	No NAFLD (n = 93)	NAFLD without NASH (n = 86)	NASH (n = 23)
**Age** (mean years, SD)	54.3 (9.9)	53.3 (11.1)	53.9 (8.3)
**Male gender** (%)	71 (76.3)	68 (79.1)	18 (78.3)
**Ethnicity** (%)			
White/Caucasian	42 (45.2) *	39 (45.3) *	16 (69.6) *
Black non Hispanic	35 (37.6) *	35 (40.7) *	4 (17.4) *
Others	16 (17.2)	12 (14.0)	3 (13.0)
**MSM** (%)	43 (46.2)	38 (43.9)	12 (52.2)
**IDU** (%)	3 (3.2)	4 (4.7)	0
**Diabetes** (%)	12 (12.9) *	8 (9.3) *	7 (30.4) *
**Hypertension** (%)	25 (26.9)	20 (23.2)	9 (39.1)
**BMI** (mean Kg/m^2^, SD)	25.8 (4.8)	27.7 (4.1)	27.7 (4.5)
**Time since HIV diagnosis** (mean years, SD)	16.4 (7.5) *	14.1 (7.6) *	19.9 (7.4) *
**Detectable HIV viral load (>40 copies/mL)** (%)	4 (4.3) *	0 *	3 (13.0) *
**Nadir CD4 count**	279.5 (187.8)	263.9 (223.4)	299.5 (255.4)
**On ART** (%)	84 (90.3)	78 (90.7)	21 (91.3)
**Current ART regimen** (%)			
PI	40 (43.0)	31 (36.0)	7 (30.4)
NNRTI	33 (35.5)	28 (32.6)	11 (47.8)
NRTI	72 (77.4)	73 (84.9)	20 (87.0)
Integrase inhibitor	27 (29.0) *	29 (33.7) *	15 (65.2) *
**Platelet count** (mean 10^9^/L, SD)	211.3 (64.5)	214.5 (59.0)	237.3 (74.7)
**AST** (mean U/L, SD)	25.3 (8.0) **	23.4 (7.0) **	50.6 (27.3) **
**ALT** (mean U/L, SD)	26.4 (12.0) **	26.8 (12.5) **	66.8 (33.9) **
**GGT** (mean U/L, SD)	39.2 (33.5) *	44.4 (38.3) *	66.4 (70.4) *
**HOMA-IR** (SD)	2.8 (3.6) *	3.4 (3.5) *	6.2 (3.7) *
**Total Cholesterol** (mean mmol/L, SD)	4.7 (1.1)	5.0 (1.0)	4.9 (1.6)
**LDL cholesterol** (mean mmol/L, SD)	2.6 (0.8)	2.8 (0.9)	2.6 (1.4)
**HDL cholesterol** (mean mmol/L, SD)	1.3 (0.5) *	1.2 (0.6) *	1.0 (0.3) *
**Triglycerides** (mean mmol/L, SD)	1.7 (1.0) *	2.0 (1.5) *	3.8 (5.3) *
**LSM** (mean kPa, SD)	4.6 (1.2) **	5.4 (2.6) **	10.2 (5.6) **
**APRI** (SD)	0.37 (0.15) **	0.36 (0.33) **	0.73 (0.63) **

No NAFLD was defined as CAP <248 dB/m; NAFLD without NASH was defined as CAP ≥ 248 dB/m and CK-18 < 246U/L; NASH was defined as CAP ≥ 248 dB/m and CK-18 > 246U/L. Continuous variables are expressed as mean (SD) and categorical variables were presented as numbers (%).

* p < 0.05

** p < 0.001.

p-values refer to T-test or chi-squared test between patients with NASH (CK-18 > 246 U/L and CAP >248 dB/m) and those with NAFLD but without NASH or those with no NAFLD and are considered significant when < 0.05. HOMA-IR was evaluated in 140 patients.

Abbreviations; ALT, alanine aminotransferase; APRI, AST-to-Platelets Ratio Index; AST, aspartate aminotransferase; BMI, body mass index; CAP, controlled attenuated parameter; ART, antiretroviral therapy; GGT, gamma-glutamyl transpeptidase; HIV, human immunodeficiency virus; HDL, high-density lipoprotein cholesterol; HOMA-IR, homeostasis model for assessment of insulin resistance; IDU, injection drug use; IU, international units; LDL, low-density lipoprotein cholesterol; LSM, liver stiffness measurement; MSM, men who have sex with men; NNRTI, non-nucleoside reverse transcriptase inhibitor; NRTI, nucleoside reverse transcriptase inhibitor; PI, Protease Inhibitors; SD, standard deviation; TE, transient elastography.

### Correlation of CK-18 levels with metabolic and biochemical parameters

The mean CK-18 levels in the whole study population was 122.5 (SD 143.2) U/L. CK-18 levels showed a significant positive correlation with ALT (r = 0.64, p<0.001), AST (r = 0.74, p<0.001), and GGT (r = 0.37, p<0.001). In addition, CK-18 levels correlated positively with triglycerides (r = 0.48, p<0.001) and HOMA-IR (r = 0.24, p = 0.03), and negatively with HDL cholesterol (r = - 0.15, p = 0.05). A positive correlation with LSM (r = 0.46, p<0.001) and APRI was also found (r = 0.51, p<0.001). CK-18 levels did not correlate significantly with BMI (r = - 0.06, p = 0.43).

### Prevalence and predictors of NASH

**Fig 2:**
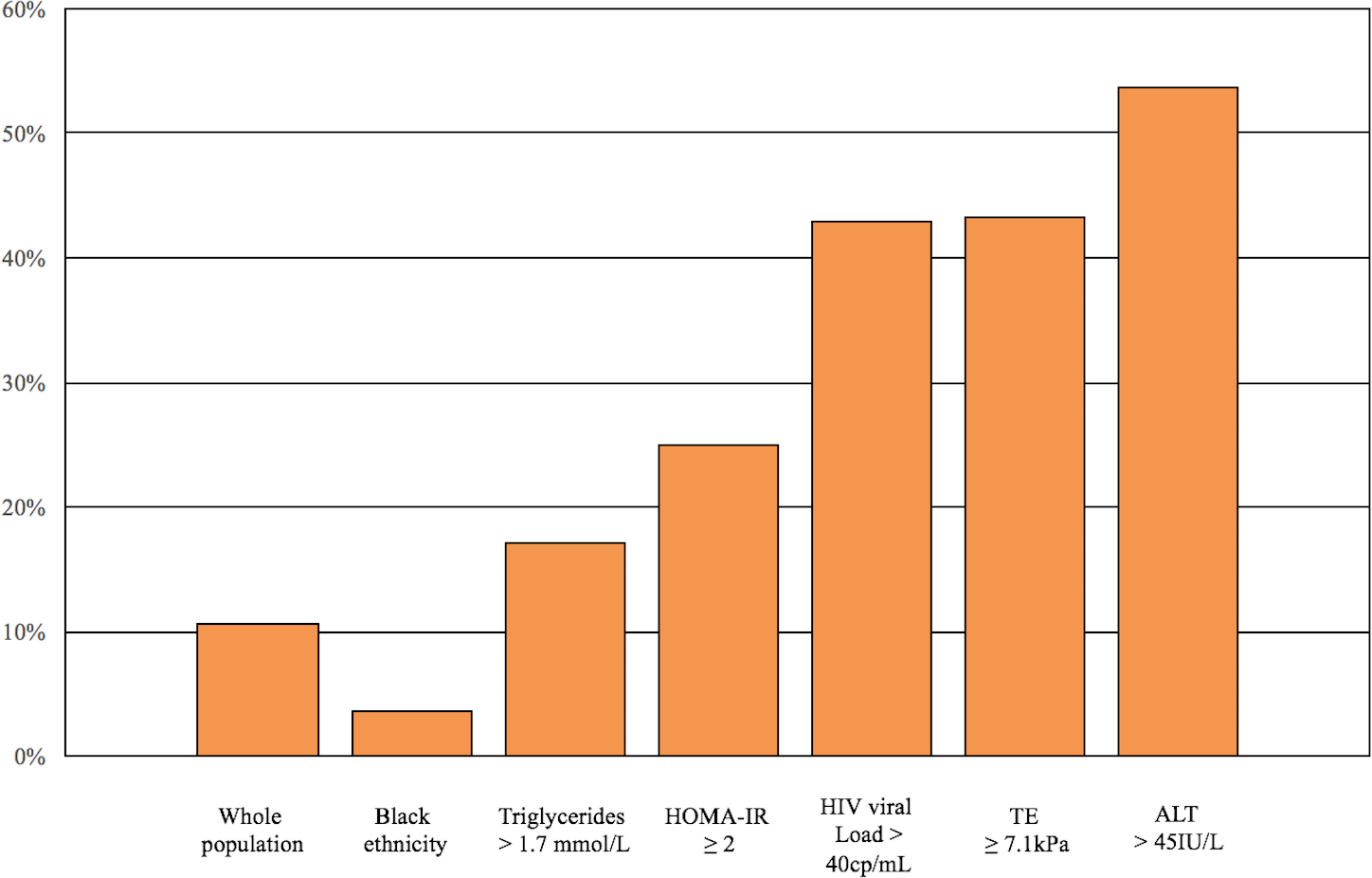
Prevalence of NASH (CAP ≥ 248 dB/m and CK-18 >246 U/L) according to patients’ characteristics.

**Fig 3:**
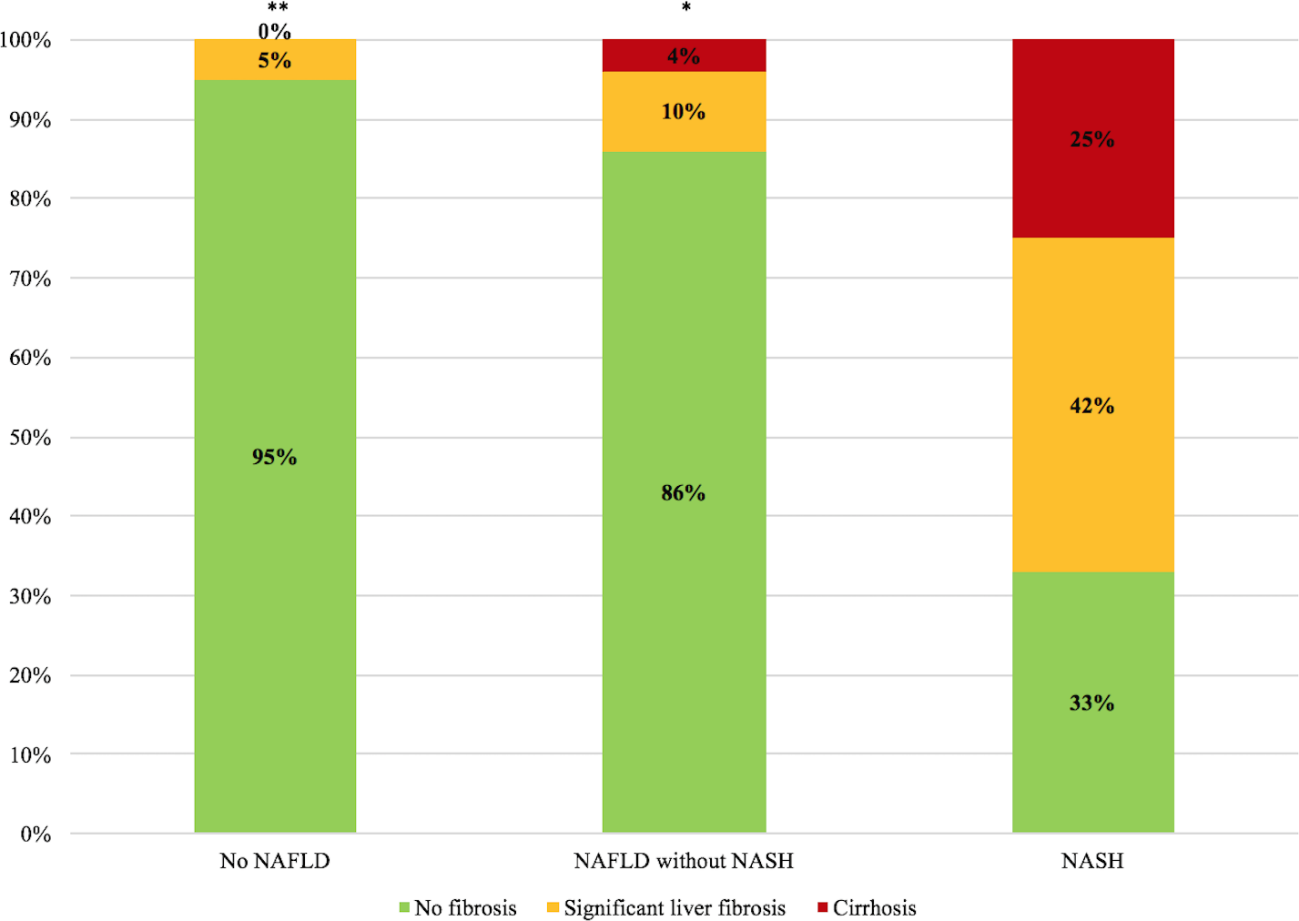
Prevalence of significant liver fibrosis (F2-3) and cirrhosis (F4) by NAFLD and NASH status. * p < 0.05; ** p < 0.001. p-values refer to chi-square test between patients with NASH (third column) and those with NAFLD but without NASH (second column) or those with no NAFLD (first column) and are considered significant when < 0.05.

Twenty-three patients had NASH, accounting for a prevalence of 11.4%. Table 1 compares demographic, clinical, biochemical, and virological characteristics of patients without NAFLD, patients with NAFLD but without NASH, and patients with NASH. Patients with NASH were more likely to be of white/Caucasian ethnicity, to have diabetes and detectable HIV viral load, they had longer time since HIV diagnosis and they were more likely to be on integrase inhibitors. They also had higher AST, ALT, GGT, and triglyceride levels. As indicated in Fig 2, the prevalence of NASH was 3.6% in patients of Black ethnicity, 17.1% in patients with hypertriglyceridemia, 25% in patients with insulin resistance, 42.9% in patients with detectable HIV viral load, 43.3% in patients with LSM ≥7.1kPa, and 53.6% in patients with an elevated ALT (>45U/L). Patients with NASH had higher prevalence of significant liver fibrosis and cirrhosis than those without it (Fig 3). We characterized also 12 patients with NAFLD without NASH who presented with significant liver fibrosis. As reported in Table 2, patients with NAFLD without NASH and significant liver fibrosis were more frequently of Caucasian ethnicity, had higher ALT and higher HOMA as compared to those without liver fibrosis.

**Table 2:** Selected demographic, clinical, and biochemical, characteristics of 86 patients with NAFLD without NASH and univariate analysis by presence of significant liver fibrosis.

Variable	Significant liver fibrosis (n = 12)	No significant liver fibrosis (n = 74)
**Age** (mean years, SD)	55.7 (10.1)	52.1 (9.32)
**Male gender** (%)	9 (75.0)	(81.9)
**White/Caucasian Ethnicity** (%)	10 (83.3) *	35 (47.3) *
**Diabetes** (%)	2 (16.7)	6 (8.1)
**BMI** (mean Kg/m^2^, SD)	27.3 (2.6)	27.7 (4.3)
**Time since HIV diagnosis** (mean years, SD)	15.5 (8.9)	13.9 (7.5)
**Nadir CD4 count**	320.5 (329.3)	251.6 (200.4)
**Current ART regimen** (%)		
PI	5 (41.7)	26 (35.1)
NNRTI	3 (25.0)	27 (36.5)
NRTI	10 (83.3)	61 (82.4)
Integrase inhibitor	3 (25.0)	23 (31.1)
**Platelet count** (mean 10^9^/L, SD)	208.2 (57.9)	216.4 (59.7)
**ALT** (mean U/L, SD)	35.8 (17.3) *	25.5 (11.2) *
**HOMA-IR** (SD)	4.5 (3.8) *	3.1 (3.4) *
**Total Cholesterol** (mean mmol/L, SD)	4.9 (1.1)	5.0 (1.0)
**Triglycerides** (mean mmol/L, SD)	1.5 (0.6)	2.1 (1.6)

Significant liver fibrosis was defined as LSM >7.1 kPa. Continuous variables are expressed as mean (SD) and categorical variables were presented as numbers (%).

* p < 0.05.

p-values refer to T-test or chi-squared test between patients with and without significant liver fibrosis. HOMA-IR was evaluated in 59 patients.

Abbreviations; ALT, alanine aminotransferase; AST, aspartate aminotransferase; BMI, body mass index; ART, antiretroviral therapy; GGT, gamma-glutamyl transpeptidase; HIV, human immunodeficiency virus; HOMA-IR, homeostasis model for assessment of insulin resistance; IDU, injection drug use; IU, international units; LSM, liver stiffness measurement; NNRTI, non-nucleoside reverse transcriptase inhibitor; NRTI, nucleoside reverse transcriptase inhibitor; PI, Protease Inhibitors; SD, standard deviation.

### Histologic findings in 17 patients with available liver biopsy

Liver biopsies were obtained in 17 out of 23 patients with a non-invasive diagnosis of NASH. Two patients refused to undergo the procedure, while the other two had a relative contraindication. The mean liver biopsy length was 18 (SD 5) mm. Significant liver fibrosis (stages F2-4) and cirrhosis (F4) were present in 10 (58.9%) and 3 (17.6%) cases, respectively. Grade 1 (5–33%), grade 2 (33–66%) and grade 3 (>66%) steatosis was present in 6 (35.3%), 5 (31.4%) and 6 (35.3%) cases, respectively. Liver biopsy confirmed the diagnosis of NASH in all patients identified by CK-18 and TE with CAP. LSM had a high diagnostic accuracy to diagnose significant liver fibrosis, with an AUC of 0.91 (95% CI 0.81–1.00).

### Predictors of NASH by multivariate analysis

Table 3 reports the multivariate analysis to assess predictors of NASH. The model incorporating HOMA-IR, detectable HIV viral load, and ALT had lower AIC and BIC values than other models, hence providing support for its use. HOMA-IR and ALT were independent predictors for NASH.

**Table 3:** Clinical and metabolic variables associated with NASH by univariate and multivariate analysis.

Variable	Unadjusted OR (95% CI)	Adjusted OR (95% CI)	p
**Age** (per 10 years)	1.06 (0.71–1.59)		
**Male gender** (yes vs. no)	1.18 (0.37–3.74)		
**Black Ethnicity** (yes vs. no)	0.21 (0.04–0.78)		
**Diabetes** (yes vs. no)	2.76 (0.97–7.91)		
**BMI** (per Kg/m^2^)	1.04 (0.94–1.16)		
**Time since HIV diagnosis** (per 10 years)	2.49 (1.34–4.62)		
**Detectable HIV viral load** (>40 cp/mL)	6.43 (1.34–30.94)	4.44 (0.43–45.70)	0.21
**AST** (per 10 IU/L)	4.31 (2.42–7.70)		
**ALT** (per 10 IU/L)	2.50 (1.73–3.62)	2.39 (1.50–3.79)	<0.001
**GGT** (per 10 IU/L)	1.11 (1.01–1.21)		
**HOMA-IR** (per unit)	1.19 (1.04–1.37)	1.20 (1.01–1.43)	0.03
**HDL cholesterol** (per mmol/L)	0.04 (0.005–0.31)		
**Triglycerides** (per mmol/L)	1.41 (1.06–1.88)		
**LSM** (per kPa)	1.50 (1.26–1.77)		

p-value is considered significant when < 0.05. HOMA-IR was evaluated in 140 patients.

Abbreviations; ALT, alanine aminotransferase; AST, aspartate aminotransferase; BMI, body mass index; CI, confidence interval; GGT, gamma-glutamyl transpeptidase; HDL, high-density lipoprotein cholesterol; HOMA-IR, homeostasis model for assessment of insulin resistance; LSM, liver stiffness measurement; OR, odds ratio; TE, transient elastography.

## Discussion

Individuals living with HIV are at high risk of developing serious liver diseases[1]. Previously thought to be mainly related to co-infections with hepatitis B or C virus, NAFLD has recently emerged as an important cause of liver pathology[3, 5, 12, 26]. Our study, based on a cohort of unselected HIV-infected patients without viral hepatitis co-infection or significant alcohol intake, shows that NASH diagnosed by the serum biomarker CK-18 and TE with CAP is frequent. Importantly, when available, histology confirmed the presence of NASH in all patients.

Due to the invasive nature of liver biopsy and its unclear clinical indications, data on NASH in HIV-infected patients are scarce. In our cohort, NASH was frequent, with a prevalence of 11.4%. In North America, the prevalence of NASH in the general population is 3–5%, meaning that HIV mono-infected patients may have twice the risk of developing NASH [5, 7]. By evaluating HIV mono-infected patients as part of a routine screening program, we have minimized the effect of a selection bias. The prevalence we report is a conservative estimate as we have not biopsied all patients, but only those with non-invasive evidence of the disease. Previous studies have selected high risk HIV mono-infected patients with either chronic elevation of transaminases or hepatic steatosis on ultrasound. This most likely led to an overestimation of NASH prevalence, which ranged between 53.3% and 63.6%[9, 27, 28].

Our data indicates that HIV-related NASH is both common and severe. Significant liver fibrosis (F2-3), was extremely frequent in patients with NASH, affecting 42% of cases, as opposed to only 10% and 5% of patients with NAFLD but without NASH and those without NAFLD, respectively. Furthermore, liver cirrhosis was detected in 25% of HIV mono-infected patients with NASH. Considering that the prevalence of significant liver fibrosis and cirrhosis in patients with NASH in the general population is much lower, it seems that patients with HIV have a more severe NASH phenotype[29]. This may be due to a specific pathophysiology in the context of HIV and to the presence of multiple, concurrent risk factors including metabolic dysfunction, chronic treatment with ART, and HIV itself[4]. Insulin resistance was highly prevalent in our cohort of HIV mono-infected individuals, affecting 61.4% of patients. The prevalence of insulin resistance in our cohort is similar to what was previously published in another cohort of Canadian patients co-infected with HIV and hepatitis C virus[24]. Insulin resistance was an independent predictor of NASH after multivariate analysis. Moreover, in patients with NAFLD without NASH it was associated with significant liver fibrosis. This is in agreement with previous reports where elevated HOMA-IR was associated with severity of NAFLD and NASH[30–32]. In previous studies, the presence of insulin resistance has been associated with other metabolic disturbances including glucose intolerance, hypertriglyceridemia, and excess weight[33]. More specifically in patients with HIV, the presence of insulin resistance has been related to the use of protease inhibitors (PI), and HIV-related hypogonadism. Our findings reinforce the significance of insulin resistance as a driver and potential modifiable risk factor for the prevention or reversal of steatohepatitis in HIV-infected patients[34]. This is particularly significant considering that insulin resistance may also be associated with more rapid progression of liver disease[24].

We confirmed the pathophysiological link between CK-18 fragments and NASH in HIV mono-infected patients by finding a positive correlation with insulin resistance, triglycerides, LSM, APRI and ALT. ALT was also an independent predictor of NASH on multivariable analysis. This indicates that liver enzyme abnormalities in patients with HIV and no known liver disease should prompt further investigations, including referral for TE examination to evaluate the degree of liver fibrosis.

Our study has several strengths. First, it is the first study from North America employing the biomarker of hepatocyte apoptosis CK-18 in the specific context of HIV-related NASH. Second, we included only consecutive, unselected HIV mono-infected patients without known liver disease as part of a routine screening program. This approach minimizes the risk of selection bias present in previous studies, which included patients with chronically elevated transaminases or fatty liver on imaging. Third, this is one of the very few histologic studies of NASH in HIV mono-infected patients. When available, liver histology confirmed the non-invasive diagnosis of NASH in all cases. Finally, we carefully ascertained and excluded the main causes of false positivity for LSM.

We wish to acknowledge several limitations of our study. First, we estimated the prevalence of NASH based on surrogate non-invasive methods. However, large-scale studies employing liver biopsy in HIV-infected patients are unlikely to be performed as this would be ethically questionable given the invasiveness of the procedure, costs, and lack of a clear clinical indication[35]. Indeed, 26% of our patients refused or had a relative contraindication to undergo liver biopsy. Second, liver biopsy was available only for positive NASH cases, so we could not account for false negative cases. Third, we did not examine CK-18 in a control population. Fourth, given the relative low number of outcomes, we were only able to examine a limited number of variables to avoid overfitting, a phenomenon resulting in poor predictive performance of models due to excessive fit with a limited set of data points. As a consequence, our results may in part suffer from imprecision of estimates and inability to tease out individual metabolic components and specific ART regimens that may drive the pathogenesis. Finally, HOMA-IR was not available in all patients, but its association with NASH was so strong that it persisted in all the multivariate models we ran.

In conclusion, in this first study employing CK-18 in unselected North American HIV mono-infected patients undergoing a routine clinical screening, we found that NASH is a common comorbidity. Given recent recommendations from the European Association for the Study of the Liver to screen high risk individuals for NASH, we suggest that HIV mono-infected individuals should be considered as a high risk target population owing to the high prevalence of disease[35]. Early identification of a subpopulation at higher risk for NASH, such as those with elevated ALT or a HOMA-IR ≥2, could optimize the use of local resources by prioritizing those who need further diagnostic assessment with CK-18, TE or liver biopsy. This may allow risk stratification and early initiation of cirrhosis surveillance when appropriate[2]. Moreover, it could potentially allow targeted interventions to avoid developing a progressive liver disease by ensuring adequate treatment of parameters of insulin resistance. Future longitudinal studies aimed at evaluating the impact of early diagnosis using non-invasive diagnostic tools and interventions on long-term hepatic morbidity and mortality are warranted.
